# Clinical, biochemical and molecular analysis of two infants with familial chylomicronemia syndrome

**DOI:** 10.1186/s12944-016-0254-z

**Published:** 2016-05-06

**Authors:** Yonghong Zhang, Jing Zhou, Wenxin Zheng, Zhangzhang Lan, Zhiwei Huang, Qingnan Yang, Chengbo Liu, Rui Gao, Yongjun Zhang

**Affiliations:** Xin Hua Hospital, Shanghai Jiao Tong University School of Medicine, 1665 Kongjiang Road, Shanghai, 200092 China; BGI-Shenzhen, Room 404 buiding No.11,Beishan industrial area, Beishan Road, Yantian District Shenzhen, 518083 China; MOE and Shanghai Key Laboratory of Children’s Environmental Health, Shanghai, China

**Keywords:** Familial chylomicronemia syndrome, Hypertriglyceridemia, Infancy

## Abstract

Familial chylomicronemia syndrome (FCS) is a rare autosomal recessive disease due mainly to inherited deficiencies in the proteins or enzymes involved in the clearance of triglycerides from circulation. It usually happens in late childhood and adolescence, which can have serious consequences if misdiagnosed or untreated. In the present study, we investigated two Chinese male babies (A and B), 30d and 48d in age, respectively, who have milky plasma. Clinical, biochemical, and radiological assessments were performed, while samples from the patients were referred for molecular diagnosis, including genetic testing and subsequent analysis of related genes. The fasting serum lipids of the two patients showed extreme lipid abnormalities. Through a low-lipid formula diet including skimmed milk and dietary advice, their plasma lipid levels were significantly lower and more stable at the time of hospital discharge. The genetic testing revealed compound heterozygote mutations in the lipoprotein lipase (LPL) gene for patient A and two known compound heterozygote LPL gene mutations for the patient B. FCS is the most dramatic example of severe hypertriglyceridemia. Early diagnosis and timely dietary intervention is very important for affected children.

## Background

Familial chylomicronemia syndrome (FCS) is a rare autosomal recessive disease which is a genetic defect of the intravascular lipolysis of triglyceride (TG)-rich lipoproteins. It is characterized by very severe hypertriglyceridemia, recurrent abdominal pain, episodes of acute pancreatitis, eruptive cutaneous xanthomata, and lipemic (“lactescent or milky”) plasma. Mutations causing FCS have been found in the following genes: lipoprotein lipase (LPL), apolipoprotein AV (APOA5), apolipoprotein CII (APOC2), lipase maturation factor 1 (LMF1), and glycosyl-phosphatidylinositol anchored high-density lipoprotein-binding protein 1 (GPIHBP1) [1]. It is documented in most cases with FCS due to mutations in the LPL gene and more rarely to loss of function mutations of genes encoding proteins related to LPL functioning: APOA5, APOC2, LMF1 and GPIHBP1 [2]. Familial LPL deficiency usually manifests by 10 years of age, and in 25 % occurs during infancy [3].

## Patients and methods

### Patients

The parents of the patients were given informed consent. Approval of the ethics committee of Xin Hua Hospital had been obtained before the study was initiated.

Patient A is a Chinese male baby, the second neonate of unrelated parents delivered by cesarean section after a full-term uneventful pregnancy. The birth weight was 3650 g. The baby remained clinically well. He was first noted to have lipemic serum at 30 days of age when blood drawn for a complete blood count could not be analyzed because of the turbidity of the serum. Physical examinations reported a well-developed child, with head circumference of 38.8 cm, and weighing 6000 g. In addition, the liver and spleen were both normal. However, eruptive xanthomatas was noted on the scalp, face and lower limbs. Blood chemistry showed a plasma concentration of total cholesterol and total triglyceride were abnormal (Table 1), amylase and lipase were still within the normal range. The baby had been exclusively breastfed since birth. The plasma lipid levels of his parents and brother were normal. Family history was unremarkable. The first therapeutic measure was a low-lipid formula diet including skimmed milk and dietary advice.

**Table 1:** Comparison of lipid profile for patients before and after treatment

	Patient A		Patient B		Normal range
	First day	Sixth day	First day	Seventh day	
TGD(mmol/L)	8.55	3.4	6.29	1.87	0.2-2.31
CHO(mmol/L)	32.82	1.06	1.89	1.05	3.36-6.46
HDL(mmol/L)	0.04	0.11	0.11	0.06	0.83-1.96
LDL(mmol/L)	0.17	0.16	0.14	0.22	2.07-3.1

*TGD* triglyceride, *CHO* cholesterol, *HDL* high-density lipoprotein, *LDL* low-density lipoprotein

Patient B is a Chinese male baby, who was born at 36 (+5) weeks by cesarean section, weighing 2800 g. The baby was observed for neonatal mild jaundice until 5 days of age. He was initially admitted to the hospital for choking on milk and for polypnea at 48 days of age. During sampling, the venous blood was remarkably pink-creamy colored. Physical examination was negative with no dysmorphic features or skin lesions, with satisfactory general conditions. Body fat distribution was normal. His head circumference was 35 cm and weight was 4720 g. Blood chemistry showed plasma levels of cholesterol and triglyceride were high abnormally (Table 1), amylase and lipase were still within the normal range. The liver and spleen were not enlarged. The baby was also on exclusive breastfeeding since birth. Parents were healthy with normal serum lipid and lipoprotein levels. No positive family history of hyperlipidemia was known.

Further laboratory investigations in both cases showed normal glucose and thyroid, liver, and kidney function. Cardiovascular examinations, including electrocardiogram (ECG) and baseline echocardiogram (ECHO), as well as the ophthalmoscopic examination and cerebral and abdominal ultrasound examinations were negative. A thorough history, physical examination, and laboratory workup failed to identify a clear etiology of the extremely abnormal blood lipid level, prompting genetic investigation to identify the genetic cause of the milky serum.

## Clinical and laboratory data

Clinical, biochemical, and radiological assessments were undertaken in Xin Hua Hospital. The lipid level was analyzed repeatedly and at the time of discharge from the hospital.

### Genetic analyses

Genomic DNA was extracted from peripheral blood leukocytes using standard method. A customized oligonucleotide probe (Nimblegen SeqCap EZ Solution) was designed to capture translated regions (exons, 50 bp extending in both directions) of forty-three genes for mono-dyslipidemia panel on conclusions from genome association studies, linkage studies, and the use of animal models [4]. Capture was performed according DNA broken, library preparation and hybridization. Then sequenced on Illumina HiSeq2500 Analyzers as PE 90 bp reads, providing an average depth of at least 100-fold for one sample. Sequenced reads were aligned to the GRCh37/hg19 human reference genome (UCSC Genome Browser) with the Burrows-Wheeler Aligner (BWA, v.0.6.2) [5]. SNVs and small insertions and deletions were called using Genome Analysis Toolkit (GATK, v.2.5.2). CNVs were assessed by read depth (RD).

## Results

### Clinical diagnosis and treatment

Upon admission, severe dietary triglyceride restriction but no lipid-lowering drug was used to treat the babies. Compared with the lipid profile at the time of admission, the lipid profile of patients after treatment lowered, which is summarized in (Table 1).

### Molecular diagnosis

**Fig. 1:**
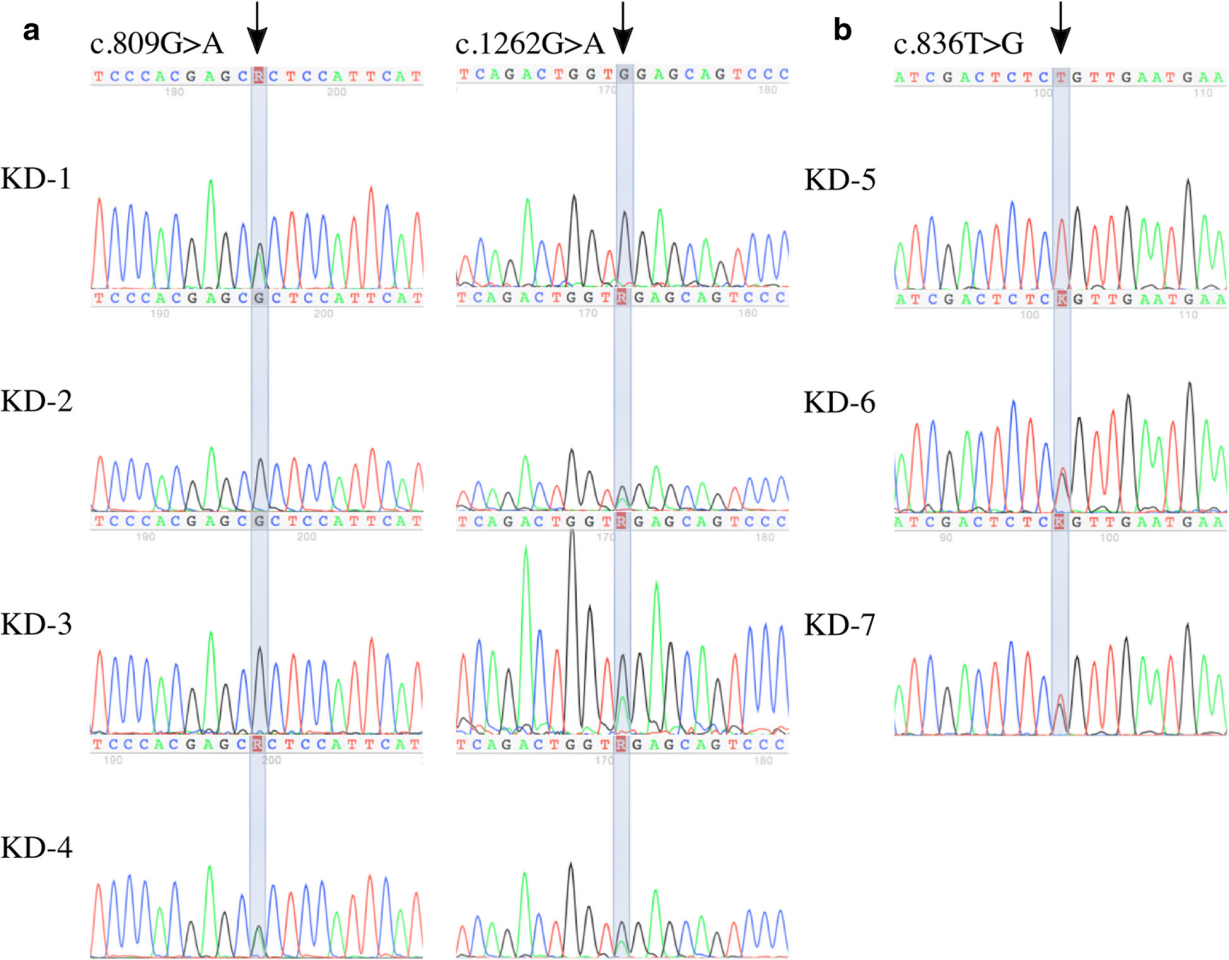
Partical nucleotide sequence of LPL gene in the probands. **a** KD-4 (proband A) was found compound heterozygosity of p.Arg270His and p.Trp421* mutations on LPL gene. Left: Chromatogram of DNA sequence analysis of LPL gene mutations. Arrow indicates the mutation site. KD-4 and KD-1 (father of proband A) were heterozygous for c.809G > A, KD-2 (mother of proband A) and KD-3 (brother of proband A) were normal.Right: KD-2, KD-3, KD-4 were heterozygous for c.1262G > A and KD-1 was normal. **b** Chromatogram of DNA sequence analysis of LPL gene mutations. Arrow indicates the mutation site. KD-7 (proband B) and KD-6 (father of proband B) were heterozygous for c.836T > G, KD-5 (mother of proband B) as normal

**Fig. 2:**
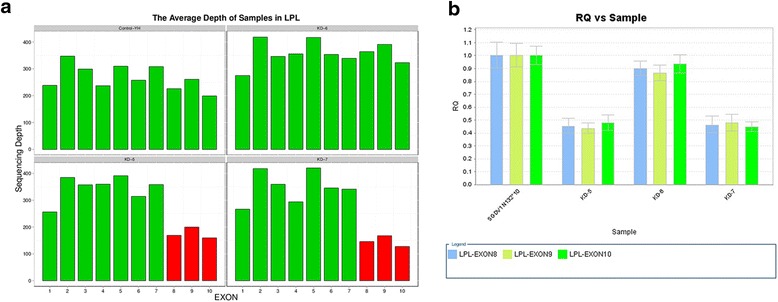
A missense mutation and a large fragment deletion in proband B. **a** The average RDs of all exons in LPL. Upper left: The average RDs of all exons in LPL from NGS showing normal sequence from a control individual. Upper right: The average RDs of all exons in LPL from KD-6 (father of proband B) showing no mutation. Bottom Left: The average RDs of all exons in LPL from KD-5 (mother of proband B) showing that there is a large fragment deletion (exon8-exon10). Bottom Right: The average RDs of all exons in LPL from KD-7 (proband B) showing that there is a large fragment deletion (exon8-exon10). **b** Sgdv1n132*10 was the control of QPCR for exon8-exon10. The result showing that KD-6 was normal, KD-5 and KD-7 were large fragment deletion carriers

We performed target region capture sequencing of the mono-dyslipidemia panel on two pedigrees to identify the genetic cause of the milky blood. For all subjects, the overall coverage of the target region was above 97 % for a minimum depth of 30X and the average sequencing depth was above 400-fold. Variants on genes known to cause chylemia (LPL, APOC2, APOA5, LMF1, and GPIHBP1) were first selected and interpreted. In the case of patient A, genetic testing revealed compound heterozygote mutations (c.809G > A p.Arg270His, c.1262G > A p.Trp421*) in the LPL gene. And a pedigree study shows that the proband’s father was carrier of c.809G > A (p.Arg270His) mutation, meanwhile his mother and brother were carriers of c.1262G > A (p.Trp421*) mutation (Fig. 1a). For patient B, genetic testing revealed two known compound heterozygote LPL gene mutations: a missense mutation (c.836 T > G p.Leu279Arg) and a large fragment deletion (deletion of exon8-exon10) (Fig. 2a). Pedigree analysis shown that the c.836 T > G (p.Leu279Arg) mutation was from his father and the large fragment deletion was from his mother. The missense mutation of patient B was confirmed by Sanger sequence (Fig. 1b). The large fragment was configured by qPCR (Fig. 2b).

## Discussion

FCS is defined as severe hypertriglyceridemia and fasting accumulations of chylomicrons in plasma, and the risk of recurrent and potentially fatal pancreatitis and other complications [1]. FCS is characterized by severe hypertriglyceridemia with episodes of eruptive cutaneous xanthomas, abdominal pain, hepatosplenomegaly, recurrent acute pancreatitis, and lipemia retinalis [6]. It usually manifests in late childhood and adolescence, but evidence suggests that presentation during infancy can be hetergeneous. It may include other signs such as anemia, jaundice, irritability, diarrhea, and pallor. These manifestations vary in time and severity. Ahmad reported a case about an infant attacked by FCS who suffered from severe complications, which highlights the need to pursue a genetic evaluation in the absence of secondary causes of severe hypertriglyceridemia in infants [7]. Besides the biochemical tests, genetic testing used on the susceptible population with positive family history of hyperlipidmia could enhance early diagnosis. In addition, close clinical follow-up and laboratory monitoring could be performed to get information about the level of lipids, and thus the occurrence of some complications would be detected without delay. APOC3 is a glycoprotein that is synthesized principally in the liver and associated with apoB-containing lipoproteins and HDL, which has been reported to be a potent inhibitor of the activation of LPL [8]. Volanesorsen is an antisense inhibitor of APOC3 synthesis, which has been extensively evaluated in several animal models on different diets in Phase 1 and Phase 2 clinical trials, and is currently being evaluated for triglyceride reduction in two Phase 3 randomized, placebo-controlled trials in patients with FCS and familial partial lipodystophy [9]. There are some other lipid-lowering agents such as DGAT1 inhibitor pradigastat evaluated in FCS patients [10]. For the treatment, there was increasing evidence suggesting the effectiveness and short-term safety of the triglyceride-lowering agents in children similar to those in adults. Long-term studies are still needed to ensure the safety and effectiveness of these agents in children [11]. In our cases, the babies were switched to dietary restraints with no lipid-lowering drug and the level of lipids soon decreased. If the lipid-lowing agents and an extremely low-fat diet could not be enough to reduce the concentrations of chylomicrons and triglyceride, nonpharmacological approaches based upon plasma-exchange (PEX) can remove triglyceride from plasma to improve the clinical conditions of patients with sHTG [2]. Timely dietary modification could improve the prognosis and maintain a normal lifestyle for affected children, by reducing the risk of some complications.

In our cases, genetic investigation could help to identify the etiology of infants with milky serum for the heterogeneity of FCS in the young group. For patient A, the genetic testing has revealed compound heterozygote mutations (c.809G > A p.Arg270His, c.1262G > A p.Trp421*) in the LPL gene (Fig. 1a). Both of the mutations were well-studied. A patient with a homozygous R270H in the LPL has been previously described [12]. Expression studies in COS-1 cells showed that the product of the mutated LPL (243 amino acids as opposed to 270 in our case) had a marked reduction in expressed mass in media and cell homogenate (24 % and 61 %). Additionally, it had no measurable level of lipolytic activity in cells or medium [13]. The nonsense mutation (c.1262G > A p.Trp421*) changes Tryptophan 421 to a stop codon (W421X), which is predicted to truncate LPL from its expected 448 AA in the mature protein to 421 AA. In vitro studies showed that Trp421 is important for the binding of lipoprotein lipase to lipoproteins [14]. For patient B, genetic testing revealed two known compound heterozygote LPL gene mutations: a missense mutation (c.836T > G p.Leu279Arg) and a large fragment deletion (deletion of exon8-exon10) (Fig. 2a). In vitro site-directed mutagenesis and expression in COS cells showed that Leu252Arg mutant LPL was associated with reduced LPL mass (32 % of the normal) and no LPL catalytic activity (0 %) [15]. A patient with the homozygous mutation of exon8-exon10 deletion has been described previously [16]. Pedigree analysis showed that the c.836T > G (p.Leu279Arg) mutation was from his father and the large fragment deletion was from his mother (Fig. 1b). The large fragment was configured by qPCR (Fig. 2b).

## Conclusions

We should pay more attention to the early diagnosis of the infants with FCS. Severe and timely dietary triglyceride restriction is one of the most effective treatment modalities for affected children. The patients should be observed with close follow-ups in future.

## Abbreviations

APOA5: apolipoprotein AV
APOC2: apolipoprotein CII
CHO: cholesterol
ECG: electrocardiogram
ECHO: baseline echocardiogram
FCS: familial chylomicronemia syndrome
GPIHBP1: glycosyl-phosphatidylinositol anchored high-density lipoprotein-binding protein 1
HDL: high-density lipoprotein
LDL: low-density lipoprotein, plasma-exchange (PEX)
LMF1: lipase maturation factor 1
LPL: lipoprotein lipase
RD: read depth
TGD: triglyceride
